# Interaction of PLP with GFP-MAL2 in the Human Oligodendroglial Cell Line HOG

**DOI:** 10.1371/journal.pone.0019388

**Published:** 2011-05-09

**Authors:** Raquel Bello-Morales, Marta Pérez-Hernández, María Teresa Rejas, Fuencisla Matesanz, Antonio Alcina, José Antonio López-Guerrero

**Affiliations:** 1 Centro de Biología Molecular Severo Ochoa, Universidad Autónoma de Madrid/Consejo Superior de Investigaciones Científicas, Cantoblanco, Madrid, Spain; 2 Instituto de Parasitología y Biomedicina “López-Neyra”, Consejo Superior de Investigaciones Científicas, Granada, Spain; University of Houston, United States of America

## Abstract

The velocity of the nerve impulse conduction of vertebrates relies on the myelin sheath, an electrically insulating layer that surrounds axons in both the central and peripheral nervous systems, enabling saltatory conduction of the action potential. Oligodendrocytes are the myelin-producing glial cells in the central nervous system. A deeper understanding of the molecular basis of myelination and, specifically, of the transport of myelin proteins, will contribute to the search of the aetiology of many dysmyelinating and demyelinating diseases, including multiple sclerosis. Recent investigations suggest that proteolipid protein (PLP), the major myelin protein, could reach myelin sheath by an indirect transport pathway, that is, a transcytotic route via the plasma membrane of the cell body. If PLP transport relies on a transcytotic process, it is reasonable to consider that this myelin protein could be associated with MAL2, a raft protein essential for transcytosis. In this study, carried out with the human oligodendrocytic cell line HOG, we show that PLP colocalized with green fluorescent protein (GFP)-MAL2 after internalization from the plasma membrane. In addition, both immunoprecipitation and immunofluorescence assays, indicated the existence of an interaction between GFP-MAL2 and PLP. Finally, ultrastructural studies demonstrated colocalization of GFP-MAL2 and PLP in vesicles and tubulovesicular structures. Taken together, these results prove for the first time the interaction of PLP and MAL2 in oligodendrocytic cells, supporting the transcytotic model of PLP transport previously suggested.

## Introduction

The myelin sheath is an electrically insulating layer that surrounds axons in both the central and peripheral nervous systems. Oligodendrocytes (OLs) are the glial cells that produce myelin in the central nervous system (CNS) [1], [2]. The presence of myelin sheath and its discontinuities, the nodes of Ranvier, allows saltatory conduction of action potential. In the absence of myelin, the velocity of nerve impulse conduction would be pathologically slow. To form the myelin sheath, OLs wrap their processes -extensions of the plasma membrane- around the axons [3], giving rise to different membrane domains and subdomains [4]. The various subdomains of OLs plasma membrane are not separated, as it occurs with basolateral and apical domains of epithelial polarized cells. Nevertheless, the myelin composition is drastically different from that of the plasma membrane of the cell body since, similar to the apical membrane of epithelial cells, myelin sheath is rich in glycosphingolipids (GSLs) and cholesterol [5]. Therefore, although myelinating OLs do not polarize segregating typical apical and basolateral surface subdomains, they can be considered as polarized cells [6].

The formation of the myelin sheath in the CNS is a highly complex process which involves the synthesis, transport, and target of large amounts of membrane proteins and lipids by OLs [7]. During OLs differentiation, several proteins and lipids segregate to form the myelin sheath. In spite of myelin composition, typical of the apical plasma membrane of polarized cells, studies *in vitro* showed that myelin sheet biogenesis has features of basolateral traffic. In this regard, vesicular stomatitis virus G protein (VSV-G), a basolateral marker, accumulated in the myelin sheet, whereas influenza virus hemagglutinin (HA), an apical marker, accumulated in the plasma membrane of the cell body, suggesting that the myelin membrane is the target of a basolateral-type pathway [8], [9].

PLP, the major myelin protein, is an integral membrane protein with four transmembrane domains. PLP and DM20, a smaller isoform generated by alternative splicing, are the most abundant proteins in the CNS myelin, comprising the 50% of total myelin proteins [1]. PLP has been associated with the low-density CHAPS-insoluble membrane fraction in cultured OLs [10], although integration of PLP into different membrane domains is a dynamic process that depends on the trafficking stage. OLs lacking PLP are still capable of myelinating axons, although physical stability of myelin decreases, since PLP is responsible for the compaction of myelin sheaths [11]. Mutations of the PLP gene cause dysmyelinating diseases in man and animals, such as Pelizaeus-Merzbacher disease, an X-linked recessive leukodystrophy [12], [13].

Crucial points on PLP traffic have still to be elucidated regarding its transport to the myelin sheath. After its synthesis in the endoplasmic reticulum, PLP is transported to the Golgi by vesicular traffic. It is yet, not entirely clear how PLP reaches its final destination, the myelin sheath. However, several studies suggest that PLP could reach the myelin sheath indirectly via the plasma membrane of the cell body rather than directly from the Golgi. This transcytotic route is similar to that observed for many apical proteins in hepatocytes, via the basolateral surface [6], [14]. If PLP travels by transcytosis, it is tempting to suggest a feasible association of this myelin protein with MAL2, a raft protein essential for transcytosis in hepatocytes.

MAL2 [15] is a 19 kD transmembrane raft protein belonging to the MAL family of proteolipids [16]. MAL2 is expressed in several epithelial cells, such as HepG2 hepatocytes, Caco-2 intestinal cells and renal MDCK cells, as well as in different types of human tissues, including peripheral neurons [17]. MAL2 has been recently associated with synaptic vesicles containing vesicular glutamate transporter-1 [18] and has been identified as an essential component of the raft-associated machinery for basolateral-to-apical transcytosis in hepatoma HepG2 cells [19], [20]. In these cells, MAL2 was found in the subapical compartment (SAC), a cellular compartment located beneath the actin belt that surrounds the bile canaliculus. The SAC [21], [22] is an endosomal compartment equivalent to the apical recycling endosome (ARE) described in other cell types [23]. The ARE/SAC is involved in basolateral-to-apical transcytosis and the recycling of proteins to the apical surface [22], [24].

In OLs, MAL2 expression was first detected in the KG-1C oligodendroglial cell line [25]. Later, a MAL2-positive compartment was described in the murine Oli-neu and human HOG oligodendrocytes derived cell lines [26]. In these cells, the MAL2-positive compartment showed some of the main features of the ARE/SAC, such as colocalization with Rab11a and CD59, peri-centrosomal location, tubulovesicular morphology, sensitivity to disruption of the microtubule cytoskeleton with nocodazole and lack of internalized transferrin, suggesting that the MAL2-positive compartment in OLs could be a structure analogous to the ARE/SAC of epithelial cells, and might therefore play an important role in the traffic of myelin proteins during OLs polarization.

Since the role of MAL2 in the traffic of transcytotic myelin proteins could be crucial for myelination, we decided to investigate the relationship between PLP and GFP-MAL2, finding that, in these oligodendrocytic cultures, both proteins colocalized after internalization from the plasma membrane, co-immunoprecipitated and, finally, colocalized at ultrastructural level. Altogether, these results indicate an association of PLP and GFP-MAL2, providing new evidences that support previous hypotheses about the transcytotic transport of PLP to the myelin sheath and further our understanding on PLP transport machinery.

## Results

### 1. Overexpression of MAL2 does not modify localization of PLP in GFP-MAL2/HOG cells

**Figure 1:**
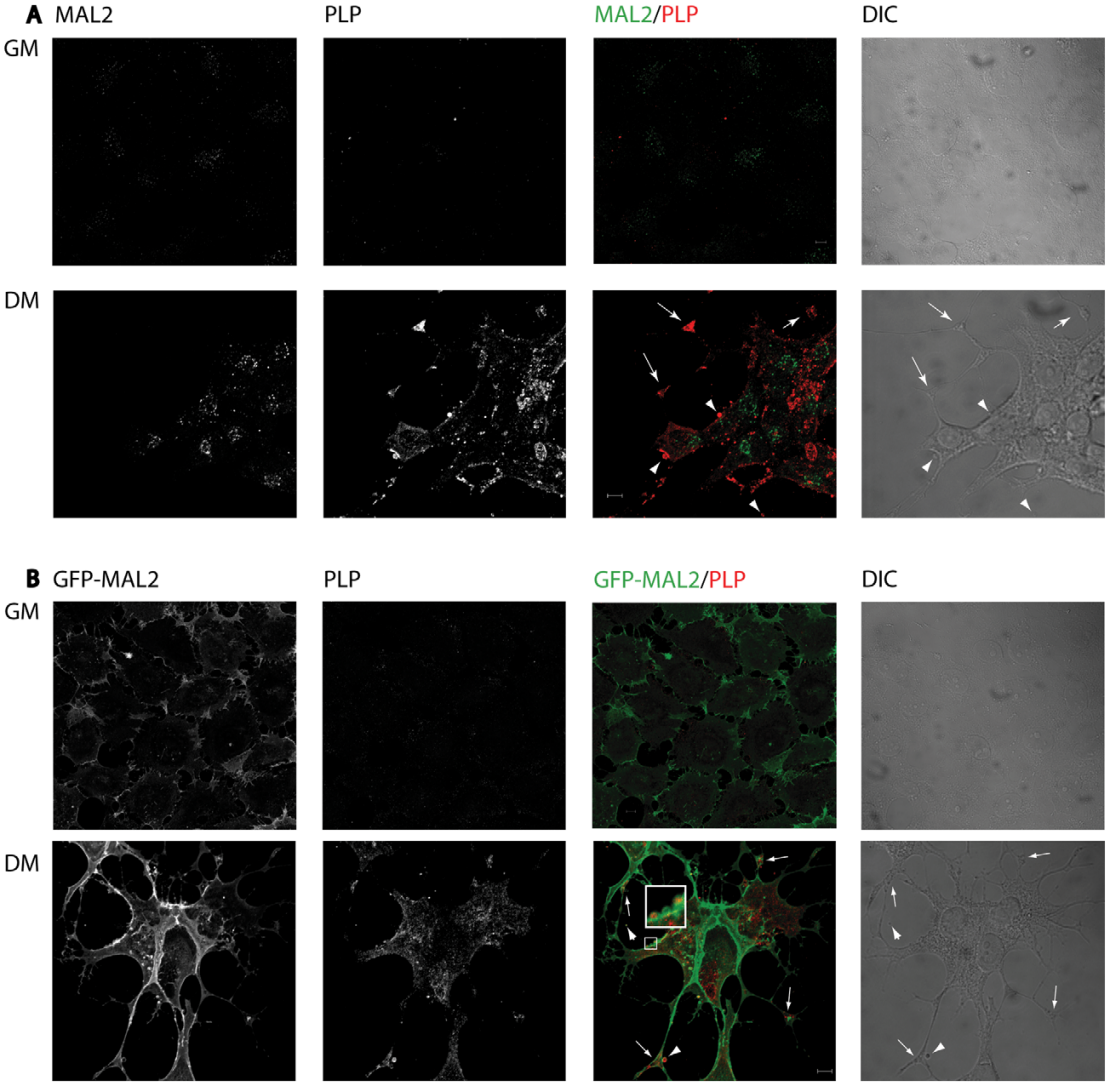
Localization of PLP in HOG and GFP-MAL2-overexpressing HOG cells. HOG cells (A) or GFP-MAL2/HOG cells (B) cultured in GM or DM were fixed and processed for confocal double-label indirect immunofluorescence analysis with anti-PLP monoclonal antibody and P239 anti-MAL2 polyclonal antibody (A) or anti-PLP antibody alone (B). Monoclonal and policlonal antibodies were detected using Alexa Fluor 555 and Alexa Fluor 488 secondary antibodies respectively. Arrows point to accumulations of PLP in myelin-like sheets. GFP-MAL2-positive vesicles containing PLP can be seen close to the plasma membrane (square and arrowheads, the square show an enlarged region). Images correspond to the projection of the planes obtained by confocal microscopy. (DIC: differential interference contrast). Scale bars  = 5 µm.

In a previous study carried out by our group, we performed the characterization of MAL2 in oligodendrocytic cells [26]. Now, we address the relationship of PLP with MAL2 proteolipid. In an initial phase, for that purpose, we used the cell line HOG stably transfected with GFP-MAL2, a construct encoding a chimera consisting of GFP fused to the amino-terminal end of MAL2. To study the localization of endogenous PLP in this GFP-MAL2 overexpressing cell line, we performed double-label indirect immunofluorescence analysis with anti-PLP monoclonal antibody and P239 anti-MAL2 antibody (Figure 1A) or PLP antibody alone (Figure 1B). Results showed that localization of PLP in GFP-MAL2/HOG cells was similar to that observed in HOG cells. Thus, culturing of cells under differentiation conditions had a similar effect on HOG and GFP-MAL2/HOG cell lines. In both lines, endogenous and exogenous MAL2 were up-regulated when cultured in differentiation medium (DM) and culturing of both cell lines in differentiation conditions resulted in an increase of PLP detection. In addition, in both cell lines PLP was detected in the plasma membrane, in vesicles scattered throughout the cytoplasm and in myelin-like sheets. In some differentiated cells the presence of MAL2 and GFP-MAL2-positive vesicles containing PLP located near the plasma membrane was also observed (Figure 1).

### 2. Partial localization of PLP in LEs/Lys

**Figure 2:**
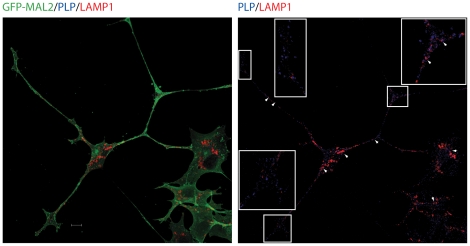
Localization of PLP and LEs/Lys in GFP-MAL2/HOG cells. Cells cultured in DM at 37°C for 2 days were fixed and processed for confocal double-labelled indirect immunofluorescence analysis with anti-PLP monoclonal and anti-LAMP1 polyclonal antibodies. Monoclonal and policlonal antibodies were detected using Alexa Fluor 647 and Alexa Fluor 555 secondary antibodies respectively. Positive vesicles for both PLP and LAMP1 can be seen (arrows), although most of PLP signal does not localize to LAMP1-positive Les/Ls. Images correspond to a confocal slice of 0.8 µm. The squares show enlarged images. Scale bar  = 10 µm.

To investigate whether, in our system, PLP is stored in late endosomes/lysosomes (LEs/Lys) after its internalization into the cells in the absence of neurons, as it occurs in other oligodendrocytic cells [27], GFP-MAL2/HOG cells cultured in DM at 37°C for 2 days were fixed and processed for confocal double-label indirect immunofluorescence analysis with anti-PLP monoclonal and anti-LAMP1 polyclonal antibodies. Results showed colocalization of both PLP and LAMP1 in some vesicles (Figure 2), although most of PLP did not localized to LAMP1-positive structures. In GFP-MAL2/HOG cells, only a small fraction of PLP is detected in LEs/Lys, suggesting that accumulation of PLP in these endosomes does not take place in this system.

### 3. PLP interacts with GFP-MAL2 in GFP-MAL2/HOG cells

**Figure 3:**
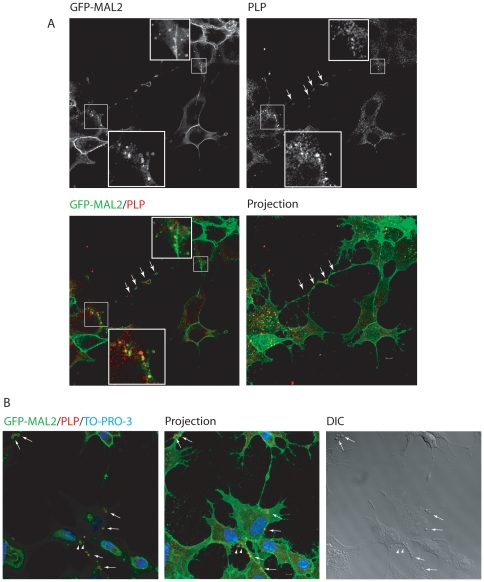
Colocalization of PLP and GFP-MAL2 in GFP-MAL2/HOG cells cultured at 37°C. Cells cultured in DM at 37°C for 2 days were fixed and processed for confocal double-labelled indirect immunofluorescence analysis with anti-PLP monoclonal antibody and Alexa Fluor 555 secondary antibody. A. Squares show vesicles positive for PLP and GFP-MAL2. Images correspond to a confocal slice of 0.8 µm, being the last one the projection of confocal planes. B. In some cells, GFP-MAL2 positive vesicles containing PLP can also be found close to the plasma membrane (arrows) and in the extracellular medium (arrowheads). Images correspond to a confocal slice of 0.8 µm. The projection of confocal planes and the DIC image are also shown. Nuclei were stained with TO-PRO-3. Scale bars  = 5 µm.

To study the relationship between PLP and GFP-MAL2, GFP-MAL2/HOG cells cultured in DM at 37°C for 2 days were fixed and processed for confocal double-label indirect immunofluorescence analysis with anti-PLP monoclonal antibody. Colocalization of PLP and GFP-MAL2 was observed in numerous vesicles throughout the cytoplasm and the myelin-like sheets (Figure 3A). Moreover, GFP-MAL2-positive vesicles containing PLP could also be found close to the plasma membrane in some cells and in the extracellular medium (Figure 3B), suggesting that GFP-MAL2-positive vesicles containing PLP could be released outside the cell.

**Figure 4:**
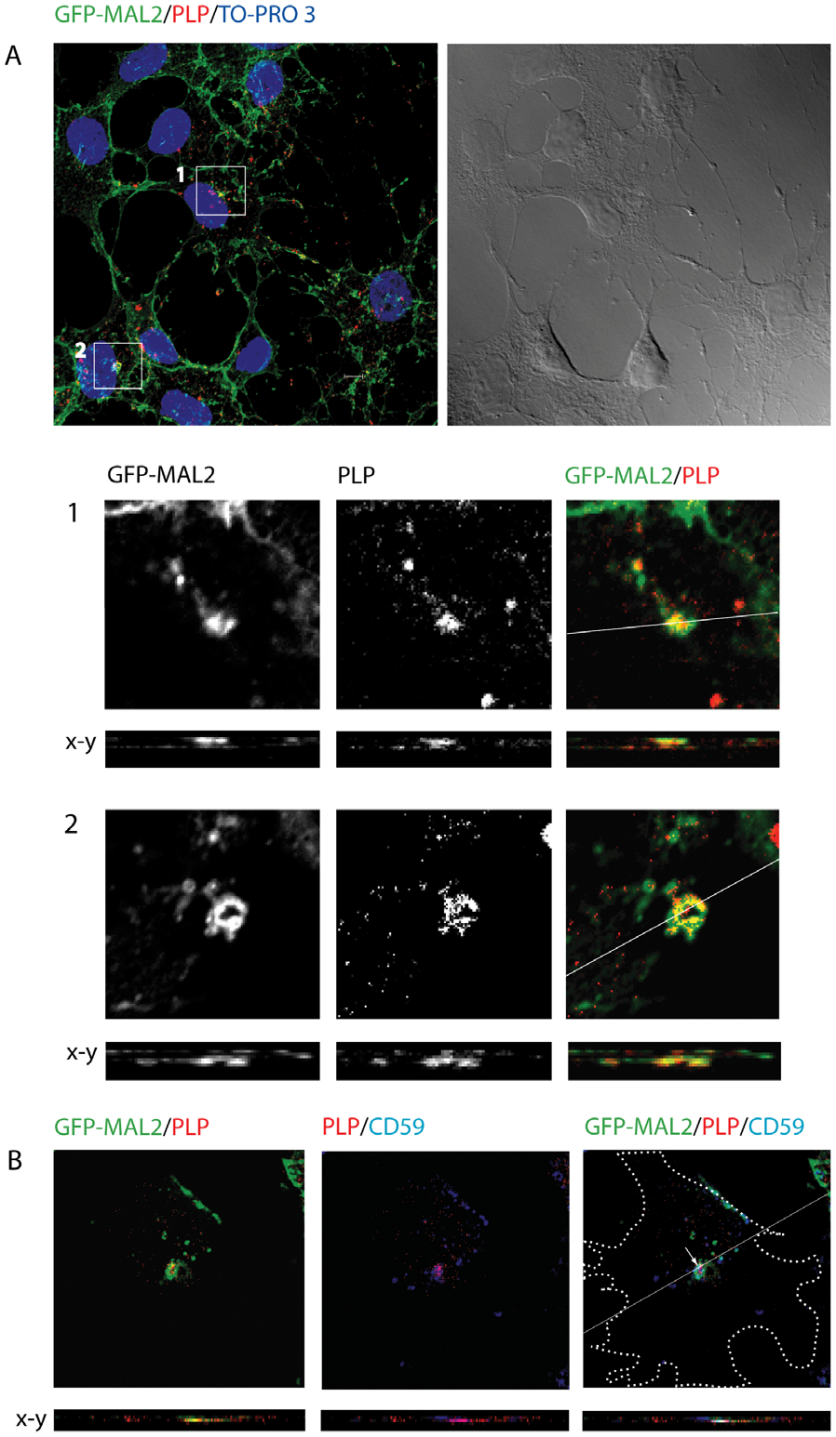
Colocalization of PLP and GFP-MAL2 in GFP-MAL2/HOG cells cultured at 18°C. Cells were cultured in DM at 37°C for 2 days, and subsequently, at 18°C for 2 h. After that, cells were fixed and processed for confocal double-label indirect immunofluorescence analysis with anti-PLP monoclonal antibody and Alexa Fluor 555 secondary antibody. A. PLP colocalizes with GFP-MAL2 in pericentrosomal compartments (squares). Image corresponds to the projection of two 0.8 µm confocal planes. Panels B and C are enlargements of the squares shown in panel A, corresponding to confocal slices of 0.8 µm. Vertical x-z sections taken at the plane indicated by the line in the corresponding horizontal section are also shown. Nuclei were stained with TO-PRO-3. D. Cells were cultured in DM at 37°C for 2 days, and then, at 18°C for 2 h. After that, cells were fixed and processed for confocal triple-labelled indirect immunofluorescence analysis with anti-PLP polyclonal and anti-CD59 monoclonal antibodies. Monoclonal anti-CD59 antibody was detected using an Alexa Fluor 647 secondary antibody. Colocalization of PLP, CD59 and GFP-MAL2 can be seen in a compartment located at the pericentrosomal region (arrow). Cell periphery is outlined. Images correspond to a confocal slice of 0.8 µm. Scale bars  = 5 µm.

Previous studies have demonstrated that exit of cargo from the ARE/SAC and further transport to the apical surface is reduced at 18°C [23], [28]. Thus, transcytosing molecules accumulate in apical recycling endosomes under such conditions. To improve conditions of colocalization of PLP with GFP-MAL2, we cultured GFP-MAL2/HOG cells in DM at 37°C for 2 days, and then, at 18°C for 120 min. Thereafter, cells were fixed and processed for confocal double-label indirect immunofluorescence analysis with an anti-PLP monoclonal antibody (Figure 4A). As results showed, under these conditions there is an increase in the number of cells exhibiting colocalization of PLP with GFP-MAL2 in a peri-centrosomal compartment.

It has been shown that CD59, a transcytotic glycosylphosphatidilinositol (GPI)– anchored protein, accumulated in the ARE/SAC of HepG2 cells and colocalized with MAL2 at 18°C [19]. In GFP-MAL2/HOG cells, CD59 colocalized at 18°C with GFP-MAL2 in the GFP-MAL2-positive compartment [26]. Accordingly, to assess the colocalization of PLP with CD59 in the GFP-MAL2-positive compartment under similar conditions, cells cultured in DM for 2 days were incubated with anti-CD59 monoclonal antibody for 30 min at 4°C, washed, and subsequently incubated for another 120 min at 18°C. Cells were then fixed and treated for triple-labelled indirect immunofluorescence analysis with an anti-PLP polyclonal antibody and Alexa Fluor 647-conjugated secondary anti–mouse antibody to detect anti-CD59 primary antibodies. Confocal images showed colocalization of GFP-MAL2, PLP and CD59 scattered in small regions of the GFP-MAL2-positive compartment (Figure 4B).

**Figure 5:**
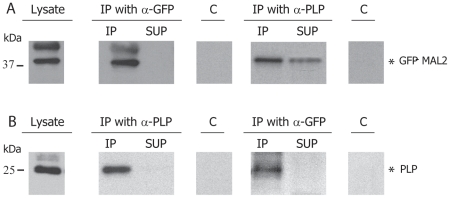
Co-immunoprecipitation of PLP and GFP-MAL2. Extracts from GFP-MAL2/HOG cells were immunoprecipitated with a monoclonal anti-PLP antibody (panel A) or with a polyclonal anti-GFP antibody (panel B) and incubated with protein A-Sepharose. Preimmune mouse or rabbit serum was used as a control (C). The whole cell lysate and the supernatants (SUP) recovered after centrifugation of the immunoprecipitates are also shown. Samples were analyzed by immunoblotting with monoclonal anti-PLP or polyclonal anti-GFP primary antibodies. Asterisks indicate height of relevant bands.

To determine whether PLP and GFP-MAL2 are in fact interacting in our system, we conducted co-immunoprecipitation studies by using a monoclonal anti-PLP or a polyclonal anti-GFP antibody. Controls included preimmune mouse or rabbit serum, the supernatants recovered after the centrifugation of the immunoprecipitate, a control without primary antibody and the whole cell lysates. SDS-PAGE analysis of the extracts immunoprecipitated with anti-PLP monoclonal antibody showed that a fraction of GFP-MAL2 coimmunoprecipitated with PLP (Figure 5), suggesting that at least a fraction of PLP is interacting with GFP-MAL2. As shown in Figure 5, the reverse assay confirmed the interaction, finding PLP in extracts immunoprecipitated with anti-GFP polyclonal antibody.

### 4. PLP colocalized with GFP-MAL2 after its internalization from the plasma membrane

**Figure 6:**
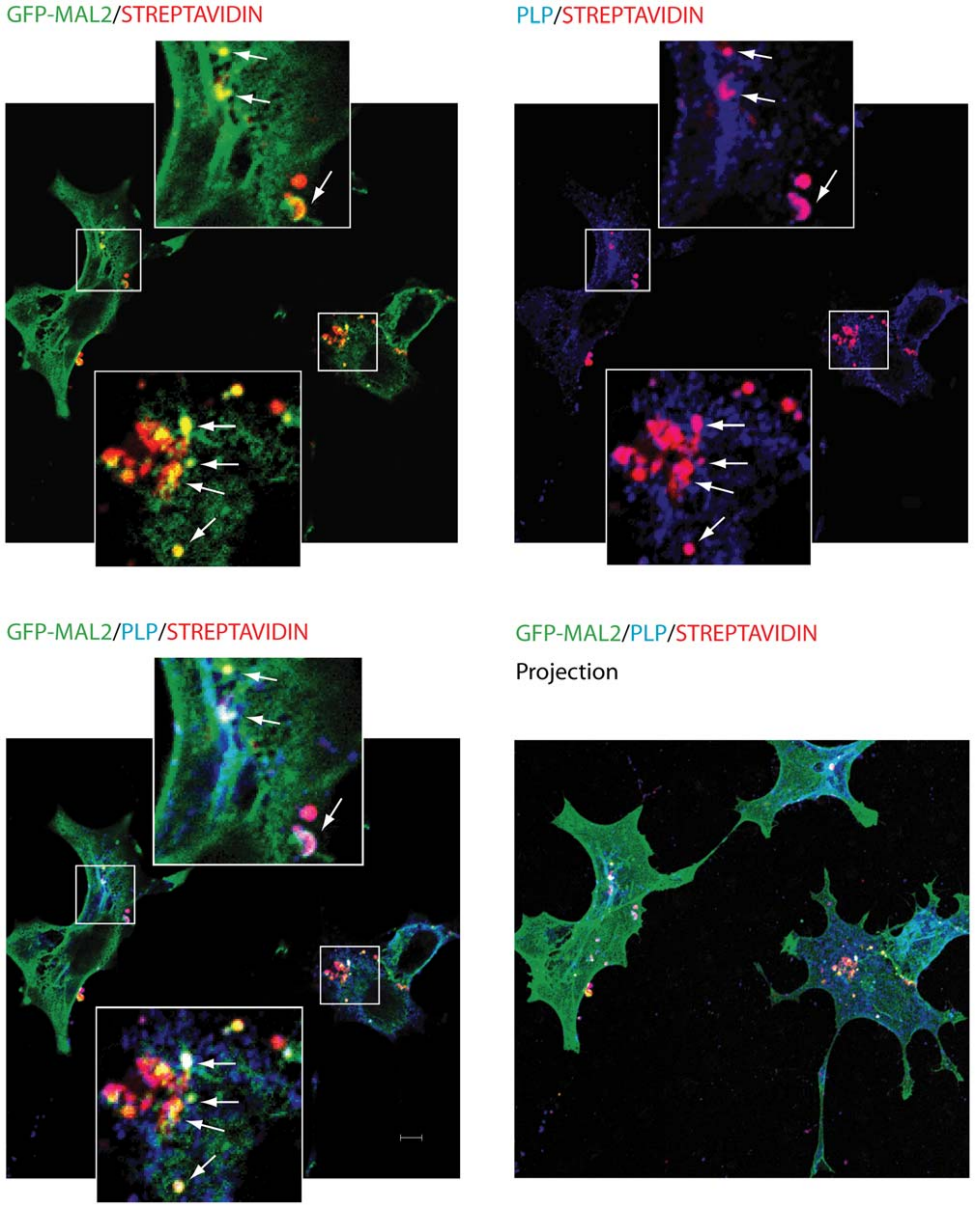
Analysis of PLP endocytosis by cell surface biotinylation. Surface proteins of GFP-MAL2/HOG cells were biotinylated at 4°C using a Sulfo-NHS ester of biotin. After biotinylation, cells were cultured in DM at 37°C for 150 min, fixed and treated for immunofluorescence analysis with anti-PLP monoclonal antibody and streptavidin-Alexa Fluor 555, to visualize biotinylated proteins. Monoclonal anti-PLP antibody was detected using an Alexa Fluor 647 secondary antibody. Images correspond to confocal slices of 0.8 µm, and the last one is the projection of confocal planes. Endocytosed streptavidin-positive proteins appear red. Arrows show endocytosed PLP (magenta) that colocalizes with endocytosed GFP-MAL2 (yellow). White vesicles correspond to endocytosed PLP colocalizing with endocytosed GFP-MAL2. Scale bar  = 5 µm.

Several studies suggest that PLP could reach the myelin sheath by indirect transport, that is, via the plasma membrane of the cell body. Therefore, if PLP travels by transcytosis, internalization from this domain will occur. After its internalization from the cell body plasma membrane, PLP would travel to the myelin sheath probably passing through other compartments involved in transcytosis, such as the MAL2-positive compartment. To study the intracellular transport of PLP from the cell surface, we performed a cell surface biotinylation assay. Cell surface proteins of GFP-MAL2/HOG cells were biotinylated at 4°C using a Sulfo-NHS ester of biotin. After biotinylation, cells were cultured in DM either for 1 hour at 4°C, or at 37°C for 20, 40, 60 and 150 min. Cells were then fixed and treated for triple-labelled indirect immunofluorescence analysis with an anti-PLP monoclonal antibody and streptavidin-Alexa Fluor 555, to visualize biotinylated proteins. Our results showed that endocytosed PLP and GFP-MAL2 colocalized in vesicles of cells incubated for 150 min (Figure 6). Vesicles positive for PLP and GFP-MAL2 were also observed in cells incubated even for 20 min, although at a lesser extent.

### 5. PLP and GFP-MAL2 colocalized with actin cytoskeleton

**Figure 7:**
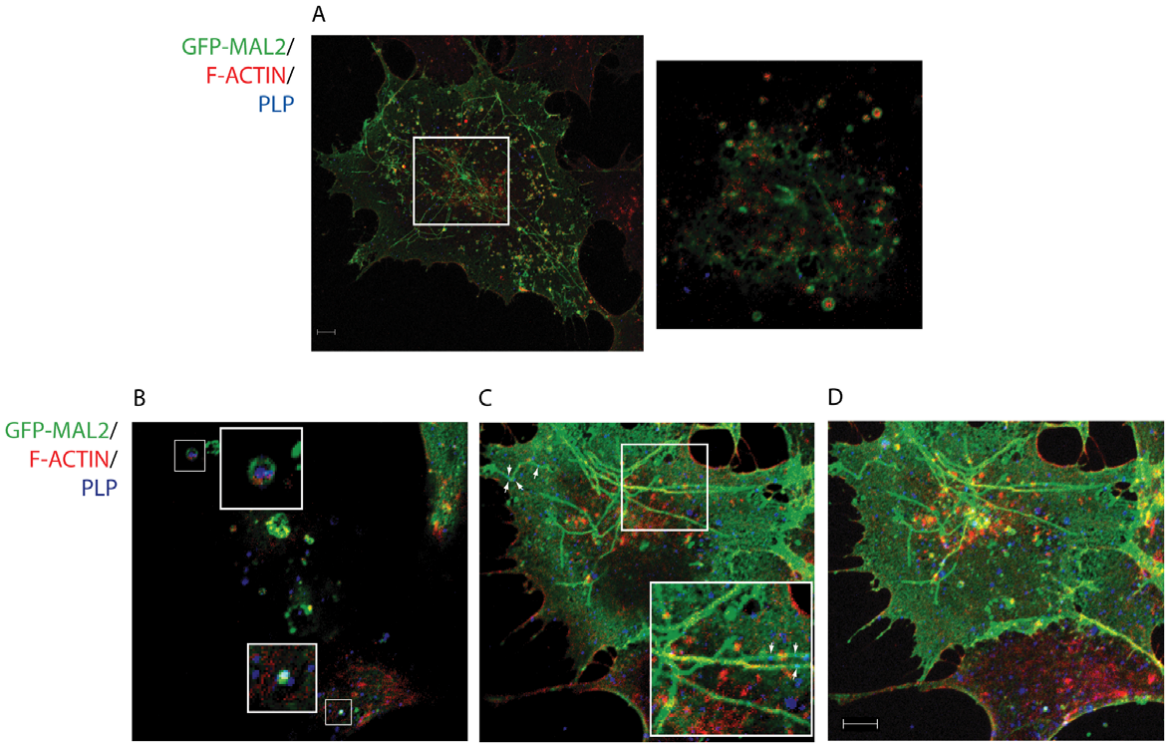
Colocalization of PLP and GFP-MAL2 with actin cytoskeleton. GFP-MAL2/HOG cells cultured in DM at 37°C for 2 days were fixed and processed for confocal triple-labelled indirect confocal immunofluorescence analysis with anti-PLP monoclonal antibody and Phalloidin-TRITC to stain F-actin. Monoclonal anti-PLP antibody was detected using an Alexa Fluor 647 secondary antibody. A. Most of GFP-MAL2-positive vesicles contain F-actin. The large image is a projection of confocal planes. The small image is an enlarged 0.8 µm confocal plane corresponding to the square. B. Colocalization of PLP, GFP-MAL2 and F-actin can be observed in some vesicles (squares). C. GFP-MAL2-positive tubular reticulum colocalizes with F-actin (squares) and PLP (arrows). Structures showing colocalization of GFP-MAL2 with F-actin appear yellow, and GFP-MAL2 with PLP appears cyan. B and C are 0.8 µm confocal planes from the same image, and D is the projection of all confocal planes corresponding to that image. Scale bar  = 5 µm.

We have previously shown that GFP-MAL2/HOG cells cultured in DM exhibited a GFP-MAL2-positive tubular reticulum arising from the peri-centrosomal region and extending towards processes [26]. Since it has been demonstrated that MAL2-positive vesicular carriers associate with actin filaments during transcytosis [29], we decided to investigate whether the GFP-MAL2-positive tubular reticulum and the vesicles positive for GFP-MAL2 were also associated with the actin cytoskeleton. In addition, we investigated whether the vesicles containing GFP-MAL2 and PLP were also associated with actin filaments. To do that, GFP-MAL2/HOG cells cultured in DM at 37°C for 2 days were fixed and processed for confocal triple-labelled indirect immunofluorescence analysis with anti-PLP monoclonal antibody and Phalloidin-TRITC to stain F-actin. Our results showed that a high proportion of GFP-MAL2-positive vesicles contained F-actin (Figure 7A). In addition, colocalization of PLP and F-actin was also observed in some GFP-MAL2-positive vesicles (Figure 7B). Herein, we also found that GFP-MAL2-positive tubular reticulum colocalized with F-actin (Figure 7C). In addition, several PLP-positive vesicles were also associated with this tubular reticulum (Figure 7C).

### 6. Electronmicroscopy (EM) immunocolocalization of PLP and GFP-MAL2

**Figure 8:**
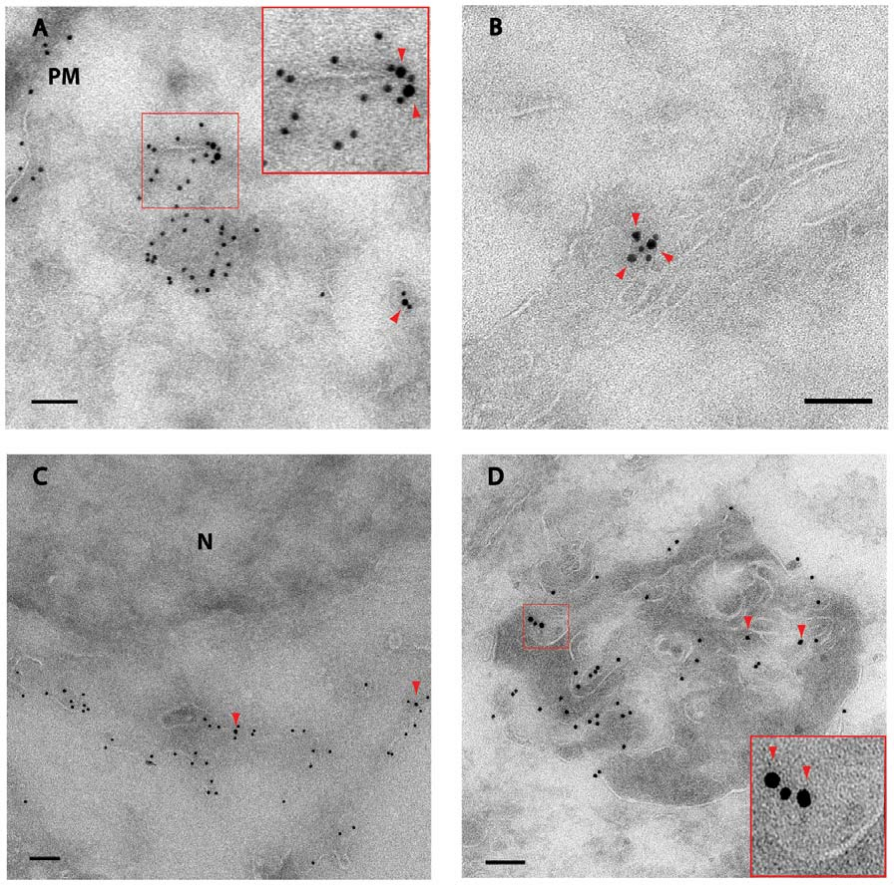
EM immunocolocalization of PLP and GFP-MAL2. Cryosections of GFP-MAL2/HOG cells cultured in DM at 37°C were stained with a rabbit polyclonal anti-GFP antibody and a mouse monoclonal anti PLP antibody. Anti-GFP and anti-PLP were detected with 10 nm protein A-gold and 15 nm anti-mouse-gold, respectively. Colocalization of PLP and GFP-MAL2 was observed in vesicles close to the plasma membrane (A); in scattered cytoplasmic vesicles (B); in tubulovesicles near the nucleus (C) and in tubulovesicular membrane-bound compartments (D). PM, plasma membrane; N, nucleus. Arrows point to 15 nm-gold particles. Scale bars  = 100 nm.

Finally, to confirm colocalization of PLP with GFP-MAL2 and to determine the ultrastructure of the organelles positive for these two proteins, we performed double-labelled immunoelectron microscopy on ultra thin cryosections. GFP-MAL2/HOG cells cultured at 37°C in DM for 2 days were processed for ultra thin cryosectioning and immuno-labeling with a rabbit polyclonal anti-GFP antibody and a mouse monoclonal anti PLP antibody. Anti-GFP and anti-PLP were detected with 10nm protein A-gold and 15nm anti-mouse-gold, respectively. Colocalization of PLP and GFP-MAL2 was confirmed in vesicles located near the plasma membrane (Figure 8A); in scattered cytoplasmic vesicles (Figure 8B); in tubulovesicles near the nucleus (Figure 8C) and in a tubulovesicular membrane-bound compartment (Figure 8D).

## Discussion

In a previous work [26], we characterized a MAL2-positive compartment in the oligodendrocytic cell lines *Oli-neu* and HOG, showing that, after differentiation, these cells up-regulate the expression of MAL2, which is accumulated in an intracellular compartment exhibiting some of the main features of the ARE/SAC, such as colocalization with Rab11a and CD59, peri-centrosomal location, tubulovesicular morphology, sensitivity to disruption of the microtubule cytoskeleton with nocodazole and lack of internalized transferrin. These results suggested that the MAL2-positive compartment in oligodendrocytic cells could be a structure analogous to the ARE/SAC seen in epithelial cells, and might therefore have an important role in the traffic of myelin proteins during oligodendrocytic polarization. Given its crucial role in transcytosis, it is reasonable to hypothesise about an involvement of MAL2 in the traffic of certain myelin proteins. This possibility prompted us to study the relationship between myelin proteins and GFP-MAL2 in our system.

Previous reports have provided data suggesting that PLP could reach myelin sheath by an indirect pathway, that is, a transcytotic route similar to that seen for many apical proteins in epithelial cells, via the basolateral surface [6], [10], [14], [27]. Motivated by this hypothesis we decided to investigate the relationship between endogenous PLP and MAL2 in our oligodendrocytic cells using the human HOG cell line. It is known that mature OLs form “myelin-like” membranes *in vitro* [5], [30]. Likewise, the HOG cell line develops myelin-like sheets when cultured under differentiation conditions and, correspondingly, these structures accumulate myelin proteins such as MAL [26] and, as we show herein, PLP. To improve MAL2 detection, we stably transfected HOG cells with GFP-MAL2, a construction already proven to be a useful tool to study MAL2 in other epithelial [20], [29] and oligodendrocytic [26] systems. Accordingly, our results show that overexpression of MAL2 does not modify significantly the localization of PLP in GFP-MAL2/HOG cells, since, in both cell lines, this proteolipid was detected in the plasma membrane, in vesicles scattered throughout the cytoplasm and in myelin-like sheets. In addition, culturing both HOG and GFP-MAL2/HOG in differentiating conditions resulted in an increase of PLP detection.

It has been demonstrated in oligodendroglial cells that, after its endocytosis, PLP is targeted to LEs/Lys, where, in absence of neuronal signals, it is stored [27], [31]. A cAMP-dependent neuronal signal triggers the retrograde transport of PLP from LEs/Lys to the surface of oligodendrocytes. In contrast with those results, in our system PLP showed a slight colocalization with LEs/Lys, since most of PLP could not be detected in LAMP1-positive endosomes. Why accumulation of PLP in Les/Lys does not take place in our system will have to be ascertained. It seems clear that the use of diverse cell systems in the different studies, with their particular features and maturation stages, is a key element probably involved or affecting the distinct behaviour of PLP during its transit to the myelin sheets.

After its synthesis, PLP is transported to the Golgi by vesicular traffic. Nevertheless, the way by which PLP reaches the myelin sheath remains to be defined. In this regard, several studies suggest that PLP could reach the myelin sheath indirectly via the plasma membrane of the cell body rather than directly from the Golgi. Therefore, if PLP travels by transcytosis, after its transport from the Golgi to the cell body plasma membrane, internalization from this domain might take place. Accordingly to this hypothesis, it has been observed that PLP present in the cell body plasma membrane is internalized and stored into LEs/Lys in absence of neuronal signals [27]. After its internalization from the cell body plasma membrane, PLP would travel to the myelin sheath possibly passing through other compartments involved in transcytosis. Therefore, after its endocytosis from this domain, it would be also feasible to find PLP associated to the MAL2-positive compartment, given that MAL2 is a raft protein essential for transcytosis in other systems. By means of confocal microscopy, we have demonstrated colocalization of PLP and GFP-MAL2 in numerous vesicles present in the cytoplasm and the myelin-like sheets of cells cultured at 37°C. In addition, culturing of cells at 18°C increased the number of cells exhibiting colocalization of PLP with GFP-MAL2.

To analyse the intracellular transport of PLP from the cell surface, we performed cell surface biotinylation. Using this method, we observed internalization of PLP from the plasma membrane and its further colocalization with GFP-MAL2, suggesting transcytosis of PLP also in our system. Some of these internalized vesicles positive for both GFP-MAL2 and PLP were localized to a pericentrosomal region, compatible with the oligodendrocytic GFP-MAL2-positive compartment analogous to the apical ARE/SAC of epithelial cells. Double-labelled immunoelectron microscopy on ultra thin cryosections also indicates a feasible interaction of PLP with GFP-MAL2. Thus, we observed colocalization of both proteins in vesicles close to the plasma membrane; in scattered cytoplasmic vesicles; in tubulovesicles near the nucleus and in a tubulovesicular membrane-bound compartment.

Since the association of MAL2-positive vesicles with actin filaments during transcytosis has been demonstrated [29], we decided to investigate whether GFP-MAL2-positive vesicles containing PLP were also associated to actin cytoskeleton. Previously, we had observed the presence of a GFP-MAL2-positive tubular reticulum in cells cultured in DM at 37°C [26]. This structure arose from the peri-centrosomal region and extended towards processes. Our present results show a large percentage of GFP-MAL2-positive vesicles containing F-actin. Moreover, colocalization of PLP and F-actin was also observed in some GFP-MAL2-positive vesicles and GFP-MAL2-positive tubular reticulum colocalized with F-actin. In addition, several PLP-positive vesicles were associated with this tubular reticulum, suggesting that actin cytoskeleton could be involved in the MAL2-mediated trancytosis of PLP in this system, as it does in other epithelial cells.

An intriguing observation was the presence of GFP-MAL2-positive vesicles containing PLP with an approximated diameter of 1.5 µm close to the plasma membrane and in the extracellular medium of GFP-MAL2/HOG cells, suggesting that these GFP-MAL2-positive vesicles containing PLP could even be released outside the cell. Similar extracellular vesicles containing PLP have also been observed in HOG cells. Large membrane vesicles, up to 1 µm of diameter, secreted by shedding from the plasma membrane, have been described in many cell types [32]. These microvesicles (MVs), different from the exosomes released upon exocytosis of multivesicular bodies, participate in important biological processes, perform a recognized role in the process of communication between cells and have significant physiological and pathological roles [33], such as inflammation, tumor progression or horizontal transfer of proteins and RNA. It has been demonstrated that cultured OLs secrete exosomes carrying PLP and other myelin proteins [34], [35], but no large MVs containing PLP had been described before in cultured OLs. However, MVs derived from oligodendrocytes have been found in the cerebrospinal fluid of multiple sclerosis patients, which highlights their pathological significance [36]. Further studies will have to determine the nature of these extracellular PLP-containing vesicles observed in our study. Despite the fact that their size is slightly larger than those of extracellular vesicles described in other cell types, due to the heterogeneity in MVs size [37], we would like to suggest that these structures could be MVs, and may be involved in cell-to-cell communication.

It has been previously demonstrated that OLs cultured in the absence of neurons express myelin genes and form myelin-like membrane sheets [30]. HOG cells have been also proved to form myelin-like membrane sheets which, in addition, accumulate myelin proteins such as MAL [26] and PLP. These membrane sheets have been previously described in several studies with primary and mixed cultures [38], [39]. Therefore, we consider that the system used in our study, oligodendroglial human HOG cell line, is a reliable system to carry out polarization studies in oligodendrocytic differentiated cells. However, further studies, with mixed primary cultures and *in vivo* systems, will be useful to corroborate these results which, anyway, are consistent with previous observations.

In conclusion, our study shows that in oligodendrocytic cells HOG, PLP and GFP-MAL2 colocalize after internalization from the plasma membrane, co-immunoprecipitate and, finally, colocalize at ultrastructural level, suggesting an association between PLP and GFP-MAL2 in this system, providing new insights into transcytotic transport of PLP to the myelin sheath.

## Materials and Methods

### Antibodies and reagents

Anti-PLP mouse monoclonal antibody MAB388, and Horseradish peroxidase-conjugated secondary anti-IgG antibodies were from Millipore (Billerica, MA, USA). Anti-PLP goat polyclonal antibody sc-18529 was from Santa Cruz Biotechnology (California, USA). Mouse monoclonal antibody to CD59 (MEM-43/5) was kindly provided by Dr V. Horejsi (Institute of Molecular Genetics, Prague, Czech Republic). P239 anti-MAL2 rabbit polyclonal antibody was kindly provided by Dr M.A. Alonso (CBMSO, Madrid, Spain). Anti-LAMP1 rabbit polyclonal antibody Ra-lamp1 was kindly provided by Dr S. Carlsson (University of Umea, Sweden). Sulfo-NHS-LC-Biotin was purchased from Thermo Scientific. Anti-GFP rabbit polyclonal serum A6455, TO-PRO-3 iodide 642/661, Alexa Fluor 488-, Alexa Fluor 647- and Alexa Fluor 594-conjugated secondary antibodies and Streptavidin-Alexa Fluor 555 were obtained from Molecular Probes (Eugene, OR, USA). Fetal bovine serum (FBS), insulin, triiodothyronine (T3), apo-transferrin, sodium selenite, putrescine, dibutyryl cyclic AMP (dbcAMP), protease inhibitor cocktail, Protein A-Sepharose, Phalloidin-TRITC and isobutyl-3,3-methyl xanthine (IBMX) were purchased from Sigma Chemical Co. (St. Louis, MO, USA). Mowiol was from Calbiochem (Merck Chemicals, Germany).

### Cell lines

The human HOG cell line, established from a surgically removed human oligodendroglioma [30] was kindly provided by Dr. A. T. Campagnoni (University of California, UCLA, USA). Cells were cultured on Petri dishes in growth medium (GM) containing Dulbecco's modified Eagle's medium (DMEM) supplemented with 10% fetal bovine serum (FBS), penicillin (50 U/mL) and streptomycin (50 µg/mL) at 37°C in an atmosphere of 5% CO_2_.

GFP-MAL2/HOG cells consisted of HOG cells stably transfected with a chimera consisting of green fluorescent protein (GFP) fused to the amino-terminal end of MAL2 (GFP-MAL2) [20], [26]. To induce maturation, cells were cultured in differentiation medium (DM) [26], containing DMEM without FBS, supplemented with antibiotics and 50 µg/ml apo-transferrin, 0.5 mg/l insulin, 30 nM triiodothyronine (T3), 30 nM sodium selenite and 16.1 mg/l putrescine. HOG cells cultured in this medium were also treated with 0.5 mM dbcAMP and IBMX (a phosphodiesterases inhibitor that reduces degradation of dbcAMP) at a final concentration of 0.5 mM.

### Immunoprecipitation and immunoblot analysis

GFP-MAL2/HOG cells monolayers were washed in cold PBS, incubated 20 min in 1 ml of cold lysis buffer (50 mM Tris–HCl pH 6.8, 150 mM NaCl, 1% Triton X-100) supplemented with 1 mM phenylmethylsulfonyl fluoride (PMSF) and Complete Mini Protease Inhibitor Cocktail, and scraped in lysis buffer to collect the lysate. Cell lysates were incubated with monoclonal anti-PLP or polyclonal anti-GFP antibodies for 2 h at 4°C with gentle mixing. As control, preimmune rabbit or mouse serum was used. A sample without primary antibody was also included. Antibody–antigen complexes were captured by incubation with protein A-Sepharose for 2 h at 4°C. Sepharose beads were collected by centrifugation at 14,000 r.p.m. for 2 min at 4°C. The supernatants were reserved for controls. Sepharose beads were washed twice in lysis buffer by centrifugation at 14,000 r.p.m. for 2 min at 4°C and, finally, immunoprecipitates were eluted by boiling in SDS-sample buffer. Samples were then subjected to SDS-PAGE in 12% acrylamide gels under reducing conditions and transferred to Immobilon-P membranes (Millipore). After blocking with 5% nonfat dry milk, 0.05% Tween 20 in PBS, blots were incubated for 1 hr at room temperature with monoclonal anti-PLP or polyclonal anti-GFP primary antibodies. After several washes with 0.05% Tween 20 in PBS, blots were incubated for 1 hr with secondary antibodies coupled to horseradish peroxidase, washed extensively, and developed using an enhanced chemiluminescence Western blotting kit (ECL, Amersham, Little Chalfont, UK).

### Immunofluorescence microscopy

Cells grown on glass coverslips were fixed in 4% paraformaldehyde for 20 min, rinsed with PBS and treated with 20 mM glycine for 5 min to quench aldehyde groups. Cells were then permeabilized with 0.2% Triton X-100, rinsed and incubated with 3% bovine serum albumin in PBS for 30 min. For double-labelled immunofluorescence analysis, cells were incubated for 1 hr at room temperature with the appropriate primary antibodies, rinsed several times and incubated at room temperature for 30 min with the relevant fluorescent secondary antibodies. Controls to assess labelling specificity included incubations with control primary antibodies or omission of the primary antibodies. After thorough washing, coverslips were mounted in Mowiol. Images were obtained using an LSM510 META system (Carl Zeiss) coupled to an inverted Axiovert 200 microscope. Confocal images were processed by FIJI-ImageJ software.

To detect accumulation of CD59 in the GFP-MAL2-positive compartment [26], GFP-MAL2/HOG cells were incubated with an anti-CD59 monoclonal antibody for 30 min at 4°C, washed, and incubated for 120 min at 18°C. Subsequently, cells were fixed and treated for immunofluorescence analysis. The antibody bound to CD59 was detected using an Alexa Fluor 647-conjugated secondary anti–mouse antibody.

### Surface protein biotinylation assay

For labelling of cell surface proteins, GFP-MAL2/HOG cells grown on glass coverslips and cultured in DM for 2 days, were washed with ice-cold DMEM and incubated for 1 hr with 0.5 mg/ml of Sulfo-NHS-LC-Biotin (Thermo Scientific) in DMEM at 4°C. Then, cells were washed with 50 mM glycine in PBS to quench free biotin. After that, cells were cultured in DM either for 1 h at 4°C, or at 37°C for 20, 40, 60 and 150 min. Finally, cells were fixed and treated for immunofluorescence analysis with monoclonal anti-PLP antibody and streptavidin-Alexa Fluor 555 (Invitrogen) to visualize biotinylated proteins.

### Immunogold-labelling EM

GFP-MAL2/HOG cells cultured at 37°C in DM for 2 days were fixed in 4% paraformaldehyde in 0.1 M sodium phosphate buffer, pH 7.4, at 4°C for 2 hour. After that, cells were incubated overnight with 8% paraformaldehyde in 0.1 M sodium phosphate buffer, pH 7.4, at 4°C. Fixed cells were washed in PBS containing 20 mM glycine and embedded in 12% gelatine, infiltrated with 2.3 M sucrose and frozen in liquid nitrogen as described [31]. Cryosections (approximately 70 nm thick) were stained with a rabbit polyclonal anti-GFP antibody and a mouse monoclonal anti-PLP antibody. Primary antibodies were detected with 10 nm protein A-gold (EM Lab, Utrecht University, the Netherlands), to detect anti-GFP, and 15 nm anti-mouse-gold (British BioCell, Cardiff, UK), to detect anti-PLP. Cryosections were examined with a JEM1010 transmission EM (Jeol, Tokyo, Japan).

## References

[1] Baumann N, Pham-Dinh D (2001). Biology of oligodendrocyte and myelin in the mammalian central nervous system.. Physiol Rev.

[2] Bradl M, Lassmann H (2010). Oligodendrocytes: biology and pathology.. Acta Neuropathol.

[3] Sherman DL, Brophy PJ (2005). Mechanisms of axon ensheathment and myelin growth.. Nat Rev Neurosci.

[4] Salzer JL (2003). Polarized domains of myelinated axons.. Neuron.

[5] Jackman N, Ishii A, Bansal R (2009). Oligodendrocyte development and myelin biogenesis: parsing out the roles of glycosphingolipids.. Physiology.

[6] Maier O, Hoekstra D, Baron W (2008). Polarity development in oligodendrocytes: sorting and trafficking of myelin components.. J Mol Neurosci.

[7] Simons M, Trotter J (2007). Wrapping it up: the cell biology of myelination.. Curr Opin Neurobiol.

[8] de Vries H, Schrage C, Hoekstra D (1998). An apical-type trafficking pathway is present in cultured oligodendrocytes but the sphingolipid-enriched myelin membrane is the target of a basolateral-type pathway.. Mol Biol Cell.

[9] Klunder B, Baron W, Schrage C, de Jonge J, de Vries H (2008). Sorting signals and regulation of cognate basolateral trafficking in myelin biogenesis.. J Neurosci Res.

[10] Simons M, Kramer EM, Thiele C, Stoffel W, Trotter J (2000). Assembly of myelin by association of proteolipid protein with cholesterol- and galactosylceramide-rich membrane domains.. J Cell Biol.

[11] Boison D, Bussow H, D'Urso D, Muller HW, Stoffel W (1995). Adhesive properties of proteolipid protein are responsible for the compaction of CNS myelin sheaths.. J Neurosci.

[12] Koeppen AH, Robitaille Y (2002). Pelizaeus-Merzbacher disease.. J Neuropathol Exp Neurol.

[13] Dhaunchak AS, Nave KA (2007). A common mechanism of PLP/DM20 misfolding causes cysteine-mediated endoplasmic reticulum retention in oligodendrocytes and Pelizaeus-Merzbacher disease.. Proc Natl Acad Sci U S A.

[14] Baron W, Hoekstra D (2010). On the biogenesis of myelin membranes: sorting, trafficking and cell polarity.. FEBS Lett.

[15] Wilson SH, Bailey AM, Nourse CR, Mattei MG, Byrne JA (2001). Identification of MAL2, a novel member of the mal proteolipid family, though interactions with TPD52-like proteins in the yeast two-hybrid system.. Genomics.

[16] Alonso MA, Weissman SM (1987). cDNA cloning and sequence of MAL, a hydrophobic protein associated with human T-cell differentiation.. Proc Natl Acad Sci U S A.

[17] Marazuela M, Acevedo A, Garcia-Lopez MA, Adrados M, de Marco MC (2004). Expression of MAL2, an integral protein component of the machinery for basolateral-to-apical transcytosis, in human epithelia.. J Histochem Cytochem.

[18] Gronborg M, Pavlos NJ, Brunk I, Chua JJ, Munster-Wandowski A (2010). Quantitative comparison of glutamatergic and GABAergic synaptic vesicles unveils selectivity for few proteins including MAL2, a novel synaptic vesicle protein.. J Neurosci.

[19] de Marco MC, Martin-Belmonte F, Kremer L, Albar JP, Correas I (2002). MAL2, a novel raft protein of the MAL family, is an essential component of the machinery for transcytosis in hepatoma HepG2 cells.. J Cell Biol.

[20] de Marco MC, Puertollano R, Martinez-Menarguez JA, Alonso MA (2006). Dynamics of MAL2 during glycosylphosphatidylinositol-anchored protein transcytotic transport to the apical surface of hepatoma HepG2 cells.. Traffic.

[21] Ihrke G, Martin GV, Shanks MR, Schrader M, Schroer TA (1998). Apical plasma membrane proteins and endolyn-78 travel through a subapical compartment in polarized WIF-B hepatocytes.. J Cell Biol.

[22] Hoekstra D, Tyteca D, van ISC (2004). The subapical compartment: a traffic center in membrane polarity development.. J Cell Sci.

[23] Apodaca G, Katz LA, Mostov KE (1994). Receptor-mediated transcytosis of IgA in MDCK cells is via apical recycling endosomes.. J Cell Biol.

[24] Maier O, Hoekstra D (2003). Trans-Golgi network and subapical compartment of HepG2 cells display different properties in sorting and exiting of sphingolipids.. J Biol Chem.

[25] Bello-Morales R, Fedetz M, Alcina A, Tabares E, Lopez-Guerrero JA (2005). High susceptibility of a human oligodendroglial cell line to herpes simplex type 1 infection.. J Neurovirol.

[26] Bello-Morales R, de Marco MC, Aranda JF, Matesanz F, Alcina A (2009). Characterization of the MAL2-positive compartment in oligodendrocytes.. Exp Cell Res.

[27] Trajkovic K, Dhaunchak AS, Goncalves JT, Wenzel D, Schneider A (2006). Neuron to glia signaling triggers myelin membrane exocytosis from endosomal storage sites.. J Cell Biol.

[28] van IJzendoorn SC, Hoekstra D (1998). (Glyco)sphingolipids are sorted in sub-apical compartments in HepG2 cells: a role for non-Golgi-related intracellular sites in the polarized distribution of (glyco)sphingolipids.. J Cell Biol.

[29] Madrid R, Aranda JF, Rodriguez-Fraticelli AE, Ventimiglia L, Andres-Delgado L (2010). The formin INF2 regulates basolateral-to-apical transcytosis and lumen formation in association with Cdc42 and MAL2.. Dev Cell.

[30] Dubois-Dalcq M, Behar T, Hudson L, Lazzarini RA (1986). Emergence of three myelin proteins in oligodendrocytes cultured without neurons.. J Cell Biol.

[31] Winterstein C, Trotter J, Kramer-Albers EM (2008). Distinct endocytic recycling of myelin proteins promotes oligodendroglial membrane remodeling.. J Cell Sci.

[32] Thery C, Ostrowski M, Segura E (2009). Membrane vesicles as conveyors of immune responses.. Nat Rev Immunol.

[33] Cocucci E, Racchetti G, Meldolesi J (2009). Shedding microvesicles: artefacts no more.. Trends Cell Biol.

[34] Kramer-Albers EM, Bretz N, Tenzer S, Winterstein C, Mobius W (2007). Oligodendrocytes secrete exosomes containing major myelin and stress-protective proteins: Trophic support for axons?. Proteomics Clin Appl.

[35] Bakhti M, Winter C, Simons M (2011). Inhibition of Myelin Membrane Sheath Formation by Oligodendrocyte-derived Exosome-like Vesicles.. J Biol Chem.

[36] Doeuvre L, Plawinski L, Toti F, Angles-Cano E (2009). Cell-derived microparticles: a new challenge in neuroscience.. J Neurochem.

[37] Yuan A, Farber EL, Rapoport AL, Tejada D, Deniskin R (2009). Transfer of microRNAs by embryonic stem cell microvesicles.. PLoS One.

[38] Hayashi T, Su TP (2004). Sigma-1 receptors at galactosylceramide-enriched lipid microdomains regulate oligodendrocyte differentiation.. Proc Natl Acad Sci U S A.

[39] Schneider A, Lander H, Schulz G, Wolburg H, Nave KA (2005). Palmitoylation is a sorting determinant for transport to the myelin membrane.. J Cell Sci.

